# To what extent do disparities in economic development and healthcare availability explain between‐province health inequalities among older people in China?

**DOI:** 10.1002/hcs2.32

**Published:** 2023-03-09

**Authors:** Sol Richardson, Zhihui Li

**Affiliations:** ^1^ Vanke School of Public Health Tsinghua University Beijing China

**Keywords:** inequalities, multilevel modelling, depression, wellbeing, disability, overweight, lung function, China

## Abstract

**Background:**

Uneven economic development has led to substantial health inequalities between Chinese provinces. The extent of, and factors underlying, between‐province health inequalities have received little attention.

**Methods:**

Data from 15,278 respondents in Wave 2 (2013) of the China Health and Retirement Longitudinal Study (CHARLS) were used to investigate inequalities among people aged ≥50 years in five health outcomes between 27 Chinese province‐level administrative units. After characterizing the between‐province differences and the relevance of province effects, proportional change in variance between unadjusted and adjusted models was calculated to determine the percentage of between‐province variance in health outcomes explained by province‐level variables including measures of economic development and healthcare availability.

**Results:**

Although province effects explained <10% of overall variance in health outcomes, they underpinned large between‐province inequalities among people aged ≥50 years. Gross Regional Product per capita was more important than doctor density in explaining between‐province variance in health outcomes, particularly depression symptoms and instrumental activities of daily living impairment.

**Conclusion:**

Policy efforts, including more equal distribution of healthcare personnel, may be warranted to reduce between‐province health inequalities.

## Graphical abstract

The results of this study, which analyzed decomposition of variance in five health outcomes between Chinese provinces using nationally representative panel data, highlight the wide health inequalities among people aged ≥50 years. For example, comparing similar individuals from randomly selected provinces, the median impact of between‐province residual heterogeneity (i.e., comparing higher and lower risk provinces) on depression symptom outcomes or instrumental activities of daily living is comparable to that of being divorced, separated, or widowed versus being married or cohabiting with a partner. Province‐level per capita economic output explained a greater proportion of the differences in these health outcomes between provinces than access to healthcare as measured by licensed doctors per capita.

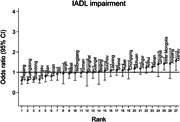

**Abbreviations**
95% CI: 95% confidence intervals
BMI: body mass index
CES‐D: Center for Epidemiologic Studies Depression Scale
CHARLS: China Health and Retirement Longitudinal Study
COPD: chronic obstructive pulmonary disease
GRP: gross regional product
IADL: instrumental activities of daily living
ICC: intracluster correlation coefficient
MOR: median odds ratio
NBS: National Bureau of Statistics (China)
OR: odds ratio
PCV: proportional change in variance
PEF: peak expiratory flow

## 1 INTRODUCTION

China has experienced rapid economic development since 1978 and a transition from a primarily agricultural to an industrial economy [1]. This process has been accompanied by rising inequalities in wealth and income from the household level to the province level, however. Economic disparities between export‐oriented coastal provinces and inland provinces in terms of gross regional product (GRP) increased markedly from around 1990 until the mid‐2000s [2]. Between‐province differences in GRP per capita and household income have since started to decline since then, however [3].

Economic changes have been accompanied by a shift in China's population age structure due to declines in both fertility and mortality, and growth in the proportion of people aged ≥50 years among the general population partially as a consequence of the One‐Child Policy [4]. At the same time, noncommunicable diseases have become a significant policy concern even since the early 1990s [5], driven by changes in diet, physical activity, smoking prevalence, environmental pollutants, and other factors [4, 6].

Economic liberalization and uneven development have contributed to increasing inequity in access to public services. Following initial economic reforms, financing of health was devolved from central to provincial governments, and rural health services organized by agricultural collectives were dissolved [7]. This has resulted in an increasing concentration of health service expenditure and utilization in more developed provinces [8], greater between‐province inequality in health resources such as health workers and beds, and wide disparities in physician pay [3, 9].

The relative extent to which inequalities in population health status is explained by economic inequalities and inequalities in healthcare access has yet to be elucidated, and both have contributed to growing between‐province health inequalities. This is particularly the case for noncommunicable diseases. Regarding specific outcomes, although prevalence of depression is comparable with other developing countries, there are wide disparities in prevalence between different regions of China [10, 11]. Prevalence of impairment in instrumental activities of daily living (IADL) has decreased over time but remains higher in rural areas [12]. Prevalences of overweight and chronic obstructive pulmonary disease have risen markedly in recent decades and vary significantly across Chinese regions [13, 14].

To my knowledge, no systematic attempt has been made to quantify the degree of between‐province health inequalities in China, or to identify variables underlying these inequalities. While previous studies have investigated province‐level effects in China, these failed to quantify either the magnitude of between‐province inequalities or the proportion of variance explained by province‐level variables [15, 16]. Partitioning of variance within a multilevel framework has been employed in studies in other contexts. For example, one study has investigated the proportion of between‐country variance in well‐being change following exit from paid employment explained by national‐level welfare policies across European countries [17].

The objectives of this study were to:

(1) Characterize prevalence of five health outcomes (depression symptoms, IADL impairment, limitation in physical functioning, overweight, and lung function impairment), and per capita economic output and doctor density, by province, and between‐province differences.
(2) Investigate the fixed‐effects individual‐ and province‐level predictors of each health outcome measure.
(3) Estimate and interpret the proportion of overall variance in each health outcome attributable to province effects, and the proportion of province effects attributable to per capita economic output and doctor density.

## 2 METHODS

### 2.1 Data sources

Data were obtained from Wave 2 (2013) of the China Health and Retirement Longitudinal Study (CHARLS); although more recent waves are currently available, data from this wave were used due to the availability of health outcomes in the “biomarkers” module (absent from Wave 4) and the low degree of missingness in important individual‐level variables. This biannual, nationally representative sample of the middle‐aged and elderly population of China, with self‐reported and objective assessments of respondents’ social, economic and health circumstances, encompasses 27 Chinese provincial‐level administrative units (provinces, municipalities, and autonomous regions) excluding Hainan, Ningxia, Shaanxi, Tibet Autonomous Region, Macau Special Administrative Region, Hong Kong Special Administrative Region, and Taiwan [18]. The analytic sample included respondents aged ≥50 years (any outcome, *n* = 15,278)^1^. CHARLS has been approved by the ethics committee of Peking University Health Science Center and all participants gave written informed consent before participation. No further ethical approval or participant consent was required for this study as it was based on a secondary analysis of existing data.

### 2.2 Variable definitions

We defined six health outcome measures, operationalized as binary variables. These included depression symptoms based on the CES‐D‐10 instrument [19], which consists of 10 Likert‐type items and yields scores ranging from 0 to 30.^2^ A cutoff score of 10, which has shown high specificity in samples of older people, was used to define probable depressive cases [20, 21]. Limitation in physical functioning^3^ and IADL impairment^4^ were defined using self‐reports of difficulties in performing physical and functional tasks. Overweight was defined using a body mass index (BMI) cutoff of ≥24, which is the typical cutoff employed in China [22], based on recordings of height and weight taken during the nurse visit. Lung function impairment was defined as having a peak expiratory flow (PEF) of <70% of an individual's expected value based on a formula including age, sex, and height [23, 24]^5^.

Two province‐level variables for 2013 were extracted from the National Bureau of Statistics (NBS) China Statistical Yearbook to measure economic development and healthcare availability [28]. GRP per capita, a province‐level measure of economic output equivalent to Gross Domestic Product per capita, was denominated in 2015 Yuan (¥) per inhabitant by increments of ¥1,000 (or $282 in 2015 Purchasing Power Parity adjusted United States Dollars) [29]. Doctor density was measured in doctors per 10,000 inhabitants. Doctors are defined as those who have passed a licencing examination and registered at a county or higher level as physicians or assistant physicians [9].

Individual‐level covariates included sex (male or female), age (years), partnership status (married and cohabiting, married but living apart, never married, or divorced, separated or widowed), ethnicity (Han or other), residence (urban or rural), health insurance (uninsured, enhanced government‐sponsored insurance, basic government‐sponsored insurance or private insurance)^6^, employment status (working or not working), quintile of gross square root equivalized household income, level of education (less than elementary school, elementary school, middle school, high school or vocational college and university), smoking status (nonsmoker or current smoker) and alcohol intake (none, once per month or >once per month). A 0–7 point index of housing quality was specified as a proxy for household wealth and based on the presence of electricity, an indoor toilet, central heating, internet, running water, sufficient living space (<2.5 people per room), and brick or concrete as primary building materials (Cronbach's *α* = 0.81).

### 2.3 Analytic methods

All analyses were performed in Stata 14 [30]. We used logistic random‐effects models for binary outcomes using the *xtmelogit* command to investigate the odds of respondents meeting the criteria for each of the six health outcome measures. Observations were nested within province‐level units and considered nonindependent due to spatial dependence. All models fitted random intercepts for each province. For models with covariate adjustment, covariates were fitted as fixed effects only. Analysis of variance components involved estimations of three random‐effects statistics: intraclass‐correlation coefficient (ICC), median odds ratio (MOR), and proportional change in variance (PCV).

ICC is defined as “the proportion of the variance explained by the grouping structure” [31]. Calculations of ICC are based on both individual‐level and area‐level variance. In multilevel logistic regression, area‐level variance is calculated on a logistic scale while individual‐level variance is on a probability scale. This study employed the latent variable method to convert individual‐level variance to the logistic scale [31].

In logistic random‐effects models for binary outcomes, ICC is calculated using the latent variable method by the following equation [32]:

(1) $$\mathrm{ICC}=\frac{V_{A}}{\left(V_{A}+3.29\right)},$$

where *V_A_* represents residual area‐level variance. The unobserved individual variable follows a logistic distribution with variance equal to π^2^/3 (3.29). The latent variable method assumes that ICC is a function only of the area‐level variance and is independent of the prevalence of the outcome.

The simulation method, proposed by Merlo et al. [33], was not used as it was considered unecessary. First, although estimation of random‐effects parameters may be biased when there are fewer than 20 area‐level units, this study employed data from 27 province‐level administrative units of China. Second, the assumption of the latent variable method that an individual's propensity to be positive for a given outcome is a continuous latent variable underlying the binary response variable was likely to have been met as all outcomes were derived from continuous measures and coded using cutoffs.

MOR, a measure of residual heterogeneity between areas, translates area‐level variance into an odds ratio (OR). It is defined as the median OR for a given outcome between the area at highest risk and the area at lowest risk when randomly selecting two areas, and is statistically independent of the prevalence of the phenomenon in question [33]. MOR can be directly compared with fixed‐effects OR estimates from the same model when considering the relevance of residual between‐area heterogeneity. The MOR is based on estimates of area‐level variance (*V_A_*) and was calculated using the following formula:

(2) $$\mathrm{MOR}=\text{exp}[\sqrt{\left(2\times V_{A}\right)}\times 0.6745].$$

In this context, 0.6745 is the 75th centile of the cumulative distribution function of the normal distribution with mean 0 and variance 1.

PCV is calculated by comparing variance estimates from an “unconditional” or “empty” intercept‐only model (without adjustment) with those from a “conditional” model (adjusted for individual‐ and/or province‐level covariates) [34]. It is defined as the percentage difference in area‐level variance between the empty and conditional models, and describes the proportion of area‐level variance explained after adjusting for (individual or groups of) individual or area‐level variables in the conditional model.

Between‐area differences can be attributable to compositional (i.e., differences in their population characteristics) and contextual factors (i.e., between‐area differences). PCV, expressed as a percentage of level‐2 variance explained by compositional and/or contextual factors, was estimated using the following formula:

(3) $$\mathrm{PCV}=\frac{\left(V_{A}-V_{B}\right)}{V_{A}}\times 100.$$

where *V_A_* represents an estimate of area‐level variance from the “empty” (unconditional) intercept‐only model without adjustment for either individual‐ or province‐level variables (Model 2 in this study) and *V_B_* residual area‐level variance from the “conditional” model (Models 3–6) with adjustment for individual‐ and province‐level covariates.

### 2.4 Descriptive analysis

We estimated the prevalence of each of the six health outcomes in 2013, both overall and by province, using cross‐sectional survey weights for individual respondents. Provinces were categorized by quartile of prevalence of each health outcome, per capita GRP, and doctor density. Individual‐level characteristics of the analytic sample were then described for each outcome.

### 2.5 Statistical analysis

Six models were fitted for each health outcome. Model 1 was an unconditional model fitted for random‐effects only and without adjustment for individual or province‐level variables. It used the full available data sample without excluding observations with missing covariate data. We then fitted another empty model for random‐effects only using the analytic sample after dropping observations with missing data without adjustment for any individual or area‐level variables (Model 2). Between‐province variance parameter estimates from Model 2 were then compared with subsequent models with adjustment for individual‐level (Model 3) and province‐level variables (Models 4–6) to calculate the percentage of between‐province variance in the empty model attributable to compositional and contextual factors. The *meresc* package was used to rescale the results of *xtmelogit* models to the same scale as unconditional intercept‐only models and allow comparison of variance components across models [35]. We analyzed complete cases only as *meresc* does not support missing data techniques such as multiple imputation.

Model 3 was fitted as a conditional model with individual‐level variables (compositional factors), and fixed‐effects OR and 95% confidence intervals (95% CI) were estimated to show associations between individual‐level variables and each health outcome. Model 4 was fitted as a conditional model with adjustment for individual‐level variables and province‐level GRP per capita while Model 5 was adjusted for individual‐level variables and doctor density. Model 6 fitted all individual level covariates in addition to both province‐level GRP per capita and doctor density. Fixed‐effects associations between province‐level variables and health outcomes, in addition to PCV compared with Model 2, were estimated for Models 4–6.

## 3 RESULTS

### 3.1 Descriptive analysis

Table 1 shows GRP per capita and doctor density by province and categorizes them by quartile. Province‐level GRP per capita ranged from ¥23,151 (Guizhou) to ¥100,105 (Tianjin). Mean GRP across the 27 provinces was ¥49,248. Mean doctor density was 2.2 doctors per 10,000 inhabitants, with a range of 1.6 (Anhui and Jiangxi) to 4.3 (Beijing). Table 2 shows the prevalence of each health outcome, in addition to GRP per capita and doctor density, by province, and categorizes provinces by quartile. There was an east‐to‐west gradient in prevalence of depression symptoms, limitation in physical functioning and IADL impairment, and a south‐to‐north gradient in overweight. Table 3 shows the individual‐level characteristics of the analytic sample for each outcome measure.

**Table 1:** China National Bureau of Statistics (NBS) estimates of gross regional product (GRP) per capita and doctor density by province in 2013

Province Total (27 provinces)	GRP per capita in 2013 (2015 ¥) Mean 49,248	Quartile	Doctors per 10,000 inhabitants in 2013 Mean 2.2	Quartile
Anhui	32,001	1st	1.6	1st
Beijing	94,648	4th	4.3	4th
Chongqing	43,223	3rd	2.0	2nd
Fujian	58,145	3rd	2.0	2nd
Gansu	24,539	1st	1.8	1st
Guangdong	58,833	3rd	2.0	2nd
Guangxi	30,741	1st	1.8	1st
Guizhou	23,151	1st	1.8	1st
Hebei	38,909	2nd	2.2	3rd
Heilongjiang	37,697	2nd	2.0	2nd
Henan	34,211	2nd	1.9	1st
Hubei	42,826	2nd	2.1	2nd
Hunan	36,943	2nd	2.0	2nd
Inner Mongolia	64,836	4th	2.5	3rd
Jiangsu	75,354	4th	2.4	3rd
Jiangxi	31,930	1st	1.6	1st
Jilin	47,428	3rd	2.5	3rd
Liaoning	61,996	3rd	2.5	3rd
Qinghai	36,875	2nd	2.3	3rd
Shandong	56,885	3rd	2.4	3rd
Shanghai	90,993	4th	2.8	4th
Shanxi	43,117	3rd	2.3	3rd
Sichuan	32,617	1st	2.1	2nd
Tianjin	100,105	4th	2.6	4th
Xinjiang	37,553	2nd	2.1	2nd
Yunnan	25,322	1st	1.7	1st
Zhejiang	68,805	4th	2.9	4th

**Table 2:** Percentage prevalence of six health outcomes by province for individuals aged 50 years and over in China Health and Retirement Longitudinal Study (CHARLS) Wave 2 (2013)

Province	Depression symptoms (CES‐D‐10)			Limitation in physical functioning			IADL impairment			Overweight			Lung function impairment		
	%	95% confidence interval (CI)	Quartile	%	95% CI	Quartile	%	95% CI	Quartile	%	95% CI	Quartile	%	95% CI	Quartile
Total (27 provinces)	31.3	30.3, 32.3		60.5	59.5, 61.5		24.6	23.8, 25.5		31.1	30.0, 32.1		51.7	50.6, 52.9	
Anhui	36.9	32.8, 41.0	3rd	67.1	63.4, 70.8	4th	29.5	25.8, 33.2	4th	25.5	0.22, 0.29	1st	59.4	54.7, 64.1	4th
Beijing	4.5	0, 9.5	1st	59.6	47.8, 71.3	2nd	20.1	8.4, 31.8	1st	85.1	0.78, 0.93	4th	42.6	27.4, 57.9	1st
Chongqing	41.8	33.5, 50.0	4th	64.7	57.8, 71.6	3rd	28.2	21.4, 35.0	3rd	31	0.24, 0.38	3rd	65.2	56.9, 73.5	4th
Fujian	35.5	29.8, 41.3	3rd	57.5	52.3, 62.6	1st	20.5	16.3, 24.6	1st	30.4	0.26, 0.35	3rd	57.6	52.0, 63.2	3rd
Gansu	52.2	45.8, 58.7	4th	66.6	61.0, 72.1	4th	39.3	33.3, 45.2	4th	27.2	0.22, 0.33	1st	58.6	52.0, 65.2	4th
Guangdong	24.6	17.8, 31.4	2nd	50.6	43.1, 58.2	1st	14.1	10.9, 17.3	1st	47	0.40, 0.54	4th	48.7	40.6, 56.8	2nd
Guangxi	34.4	29.5, 39.2	3rd	59.1	54.7, 63.5	1st	25.4	21.4, 29.4	2nd	32.1	0.28, 0.36	3rd	49	44.0, 54.1	2nd
Guizhou	35.9	27.3, 44.6	3rd	59.3	51.6, 67.0	2nd	29	21.8, 36.2	3rd	37.7	0.30, 0.45	3rd	44.7	34.6, 54.8	1st
Hebei	32.9	28.7, 37.1	2nd	64.4	60.5, 68.2	3rd	23.6	20.1, 27.0	2nd	28.2	0.24, 0.32	2nd	53	47.7, 58.2	3rd
Heilongjiang	25.8	20.1, 31.5	2nd	62.5	56.8, 68.2	2nd	26.7	20.7, 32.6	3rd	47.7	0.42, 0.54	4th	28.8	22.2, 35.4	1st
Henan	24.2	21.3, 27.1	1st	66.1	62.9, 69.3	3rd	28.8	24.9, 32.7	3rd	28.1	0.25, 0.31	2nd	45.4	41.0, 49.8	1st
Hubei	38	33.2, 42.8	4th	66.1	61.7, 70.4	3rd	27.7	23.5, 31.8	3rd	39.5	0.35, 0.44	3rd	49.8	44.4, 55.1	3rd
Hunan	35.9	31.6, 40.3	3rd	66.4	62.4, 70.4	3rd	22	18.3, 25.6	2nd	29.6	0.26, 0.34	2nd	48.9	44.0, 53.7	2nd
Inner Mongolia	32.9	28.7, 37.0	2nd	64.6	60.5, 68.7	3rd	30.5	26.6, 34.3	4th	41.3	0.37, 0.45	3rd	44.6	40.0, 49.3	1st
Jiangsu	27	22.9, 31.1	2nd	53.3	49.2, 57.5	1st	19.4	15.7, 23.1	1st	26.3	0.23, 0.30	1st	46.7	40.3, 53.1	2nd
Jiangxi	34.1	30.1, 38.1	3rd	62.8	59.0, 66.6	2nd	25.5	22.1, 29.0	2nd	30	0.27, 0.33	2nd	67.3	62.9, 71.7	4th
Jilin	24.4	19.1, 29.6	2nd	61.5	56.1, 66.8	2nd	23.5	18.9, 28.2	2nd	32	0.27, 0.37	3rd	46.8	38.0, 55.5	2nd
Liaoning	25.2	21.0, 29.5	2nd	63.9	59.4, 68.3	2nd	25.5	21.3, 29.7	2nd	27.6	0.23, 0.32	2nd	36	30.1, 41.9	1st
Qinghai	51	41.7, 60.3	4th	73.5	65.5, 81.5	4th	35.5	26.7, 44.3	4th	11.3	0.06, 0.17	1st	62.3	50.6, 74.1	4th
Shandong	22.8	20.3, 25.4	1st	52.2	49.3, 55.1	1st	17.7	15.4, 20.0	1st	22.7	0.20, 0.25	1st	41.3	37.8, 44.8	1st
Shanghai	9.8	2.7, 16.8	1st	28.6	17.7, 39.4	1st	16.1	6.6, 25.6	1st	47.6	0.36, 0.59	4th	48.5	16.9, 80.1	2nd
Shanxi	33.5	29.7, 37.3	3rd	64.2	60.8, 67.6	3rd	28.2	24.9, 31.5	3rd	24	0.21, 0.27	1st	51.9	47.8, 56.0	3rd
Sichuan	38.9	35.8, 42.1	4th	67.7	64.9, 70.4	4th	31.5	28.6, 34.4	4th	29.3	0.27, 0.32	2nd	56.6	53.2, 59.9	3rd
Tianjin	22.5	12.9, 32.2	1st	70.1	61.3, 78.9	4th	24.9	15.9, 33.9	2nd	47.1	0.37, 0.57	4th	56.2	41.9, 70.4	3rd
Xinjiang	17	8.3, 25.7	1st	88.8	81.9, 95.6	4th	46.3	34.4, 58.1	4th	44.2	0.32, 0.56	4th	55.3	42.6, 68.0	3rd
Yunnan	40.9	37.2, 44.7	4th	60	56.5, 63.4	2nd	27.1	23.9, 30.2	3rd	25.7	0.23, 0.29	1st	69.9	66.0, 73.8	4th
Zhejiang	19	15.0, 23.0	1st	44.6	40.1, 49.0	1st	14.7	11.3, 18.2	1st	27.4	0.23, 0.31	2nd	45.8	40.6, 51.0	2nd

Cross‐sectional survey weights ere applied when estimating prevalence of health outcomes.

**Table 3:** Characteristics of analytic samples for six health outcome measures, China Health and Retirement Longitudinal Study (CHARLS) Wave 2 (2013)

		Depression symptoms (CES‐D‐10) (*n* = 8204)		Limitation in physical functioning (*n* = 8874)		Instrumental activities of daily living (IADL) impairment (*n* = 8874)		Overweight (*n* = 8874)		Lung function impairment (*n* = 6692)	
Variable	Categories	*n*	%	*n*	%	*n*	%	*n*	%	*n*	%
Outcome	No	5615	68.4	3539	39.9	6929	78.1	6894	77.7	3338	49.9
	Yes	2589	31.6	5335	60.1	1945	21.9	1980	22.3	3354	50.1
Sex	Male	4154	50.6	4408	49.7	4408	49.7	4408	49.7	3300	49.3
	Female	4050	49.4	4466	50.3	4466	50.3	4466	50.3	3392	50.7
Partnership status	Married (living together)	6938	84.6	7432	83.8	7432	83.8	7432	83.8	5644	84.3
	Married (living apart)	138	1.7	153	1.7	153	1.7	153	1.7	97	1.4
	Divorced/separated/widowed	1062	12.9	1214	13.7	1214	13.7	1214	13.7	903	13.5
	Never married	66	0.8	75	0.8	75	0.8	75	0.8	48	0.7
Ethnicity	Han Chinese	7629	93.0	8254	93.0	8254	93.0	8254	93.0	6232	93.1
	Other	575	7.0	620	7.0	620	7.0	620	7.0	460	6.9
Residence	Rural	5215	63.6	5676	64.0	5676	64.0	5676	64.0	4398	65.7
	Urban	2989	36.4	3198	36.0	3198	36.0	3198	36.0	2294	34.3
Employment status	Not working	2811	34.3	3146	35.5	3146	35.5	3146	35.5	2259	33.8
	Working	5393	65.7	5728	64.5	5728	64.5	5728	64.5	4433	66.2
Health insurance	Uninsured	260	3.2	290	3.3	290	3.3	290	3.3	212	3.2
	Govt. scheme, basic coverage	6834	83.3	7425	83.7	7425	83.7	7425	83.7	5664	84.6
	Govt. scheme, enhanced coverage	977	11.9	1014	11.4	1014	11.4	1014	11.4	721	10.8
	Private or other	133	1.6	145	1.6	145	1.6	145	1.6	95	1.4
Quintile of equivalized gross household income	1 (poorest)	1647	20.1	1845	20.8	1845	20.8	1845	20.8	1409	21.1
	2	1749	21.3	1928	21.7	1928	21.7	1928	21.7	1482	22.1
	3	1672	20.4	1802	20.3	1802	20.3	1802	20.3	1388	20.7
	4	1598	19.5	1689	19.0	1689	19.0	1689	19.0	1260	18.8
	5	1538	18.7	1610	18.1	1610	18.1	1610	18.1	1153	17.2
Level of education	Less than elementary school	3724	45.4	4188	47.2	4188	47.2	4188	47.2	3170	47.4
	Elementary school	1799	21.9	1915	21.6	1915	21.6	1915	21.6	1488	22.2
	Middle school	1688	20.6	1752	19.7	1752	19.7	1752	19.7	1303	19.5
	High school	648	7.9	664	7.5	664	7.5	664	7.5	486	7.3
	Vocational college/university	345	4.2	355	4.0	355	4.0	355	4.0	254	3.8
Smoking status	Nonsmoker	3393	41.4	3622	40.8	3622	40.8	3622	40.8	2715	40.6
	Current smoker	4811	58.6	5252	59.2	5252	59.2	5252	59.2	3977	59.4
Alcohol intake	None	2266	27.6	2377	26.8	2377	26.8	2377	26.8	1794	26.8
	Once per month	606	7.4	653	7.4	653	7.4	653	7.4	508	7.6
	More than once per month	5332	65.0	5844	65.9	5844	65.9	5844	65.9	4 90	65.6
		Median		Median		Median		Median		Median	
Age	Years	61		61		61		61		61	
Housing quality	Scale (0–7)	4		4		4		4		4	

### 3.2 Fixed‐effects

Tables 4 and 5 show the fixed‐effects estimates for the associations between individual‐level variables and each health outcome (Model 3). Age and high alcohol intake (>once per month) were positively and significantly associated with higher odds of all health outcomes, except for overweight for which *p* > 0.05. Higher housing quality was negatively associated with all health outcomes except lung function impairment and overweight. Higher household income was protective against all outcomes except lung function impairment but predictive of overweight. Smoking status was associated with lung function impairment. Age was associated with lower odds of depression symptoms and overweight but positively associated with higher odds of other outcomes. Associations between other individual‐level variables and each of the six health outcomes varied.

**Table 4:** Results of conditional logistic random‐effects models for depression caseness, functional disability and instrumental activities of daily living (IADL) impairment with adjustment for individual‐level variables (Model 3)

		Depression symptoms (CES‐D‐10) (*n* = 8204)			Limitation in physical functioning (*n* = 8874)			IADL impairment (*n* = 8874)		
Variable	Categories	OR	95% confidence interval (CI)	*p*	OR	95% CI	*p*	OR	95% CI	*p*
Sex	Male	Ref			Ref			Ref		
	Female	1.55	1.35, 1.79	<0.001	1.85	1.06, 1.53	0.01	1.11	0.95, 1.30	0.195
Age	Years	0.99	0.98, 1.00	0.001	1.02	0.95, 0.97	<0.001	1.03	1.02, 1.04	<0.001
Partnership status	Married (living together)	Ref			Ref			Ref		
	Married (living apart)	1.43	1.00, 2.04	0.48	1.17	1.11, 2.57	0.014	0.91	0.57, 1.46	0.694
	Divorced/separated/widowed	1.33	1.15, 1.53	<0.001	1.05	1.13, 1.61	0.001	1.01	0.87, 1.18	0.860
	Never married	1.30	0.79, 2.12	0.299	0.82	1.16, 3.31	0.012	0.89	0.51, 1.53	0.667
Ethnicity	Han Chinese	Ref			Ref			Ref		
	Other	0.78	10.63, 0.96	0.016	1.05	0.76, 1.26	0.858	0.89	0.70, 1.12	0.311
Residence	Rural	Ref			Ref			Ref		
	Urban	0.86	0.76, 0.97	0.012	0.97	0.91, 1.24	0.445	0.87	0.76, 1.00	0.053
Employment status	Not working	Ref			Ref			Ref		
	Working	0.80	0.72, 0.90	<0.001	0.62	0.75, 1.01	0.059	0.44	0.39, 0.50	<0.001
Health insurance	Uninsured	1.00	0.77, 1.30	0.999	0.93	1.21, 2.15	0.001	0.94	0.70, 1.26	0.677
	Govt. scheme, basic coverage	Ref			Ref			Ref		
	Govt. scheme, enhanced coverage	0.93	0.76, 1.13	0.478	0.75	0.69, 1.22	0.556	0.72	0.57, 0.91	0.006
	Private or other	1.11	0.70, 1.74	0.658	0.98	0.42, 1.78	0.690	0.96	0.58, 1.56	0.856
Quintile of equivalized gross household income	1 (poorest)	1.14	0.99, 1.32	0.074	1.20	1.11, 1.61	0.002	0.99	0.84, 1.17	0.888
	2	1.17	1.01, 1.35	0.031	1.11	1.10, 1.59	0.003	1.14	0.97, 1.34	0.100
	3	Ref			Ref			Ref		
	4	0.90	0.77, 1.04	0.151	0.94	0.59, 0.90	0.003	0.87	0.73, 1.04	0.140
	5	0.69	0.58, 0.82	<0.001	0.81	0.57, 0.92	0.008	0.71	0.57, 0.87	0.001
Housing quality	Scale (0–7)	0.85	0.81, 0.89	<0.001	0.89	0.75, 0.84	<0.001	0.85	0.80, 0.89	<0.001
Level of education	Less than elementary school	Ref			Ref			Ref		
	Elementary school	0.83	0.73, 0.94	0.004	0.94	0.67, 0.93	0.006	0.70	0.60, 0.80	<0.001
	Middle school	0.80	0.69, 0.92	0.002	0.87	0.68, 0.99	0.043	0.65	0.54, 0.77	<0.001
	High school	0.78	0.63, 0.96	0.020	0.76	0.59, 1.03	0.082	0.49	0.37, 0.65	<0.001
	Vocational college/university	0.77	0.56, 1.05	0.101	0.89	0.54, 1.36	0.52	0.50	0.33, 0.74	0.001
Smoking status	Nonsmoker	Ref			Ref			Ref		
	Current smoker	1.01	0.88, 1.15	0.875	1.02	0.87, 1.24	0.665	1.01	0.87, 1.17	0.946
Alcohol intake	None	Ref			Ref			Ref		
	Once per month	1.21	0.99, 1.48	0.060	1.29	1.23, 2.03	<0.001	1.19	0.94, 1.51	0.150
	More than once per month	1.19	1.05, 1.34	0.007	1.17	1.01, 1.41	0.041	1.20	1.04, 1.39	0.015

**Table 5:** Results of conditional logistic random‐effects models for overweight and lung function impairment with adjustment for individual‐level variables (Model 3)

		Overweight (*n* = 8874)			Lung function impairment (*n* = 6692)		
Variable	Categories	OR	95% confidence interval (CI)	*p*	OR	95% CI	*p*
Sex	Male	Ref			Ref		
	Female	0.81	0.70, 0.94	0.005	1.35	1.16, 1.56	<0.001
Age	Years	1.01	1.00, 1.01	0.055	1.04	1.03, 1.05	<0.001
Partnership status	Married (living together)	Ref			Ref		
	Married (living apart)	1.56	1.09, 2.24	0.016	0.76	0.50, 1.15	0.192
	Divorced/separated/widowed	1.23	1.06, 1.43	0.006	1.01	0.87, 1.18	0.863
	Never married	1.36	0.81, 2.28	0.246	1.60	0.88, 2.90	0.122
Ethnicity	Han Chinese	Ref			Ref		
	Other	1.27	1.03, 1.56	0.025	1.23	0.99, 1.53	0.059
Residence	Rural	Ref			Ref		
	Urban	1.17	1.03, 1.32	0.016	0.85	0.75, 0.96	0.008
Employment status	Not working	Ref			Ref		
	Working	0.59	0.52, 0.66	<0.001	0.85	0.76, 0.96	0.008
Health insurance	Uninsured	1.68	1.30, 2.17	<0.001	1.05	0.80, 1.39	0.712
	Govt. scheme, basic coverage	Ref			Ref		
	Govt. scheme, enhanced coverage	1.23	1.02, 1.47	0.026	0.73	0.60, 0.89	0.002
	Private or other	1.69	1.17, 2.45	0.005	0.47	0.30, 0.75	0.002
Quintile of equivalized gross household income	1 (poorest)	1.11	0.94, 1.31	0.211	1.03	0.89, 1.20	0.680
	2	0.98	0.83, 1.15	0.764	1.00	0.86, 1.16	0.968
	3	Ref			Ref		
	4	1.03	0.87, 1.22	0.731	0.96	0.82, 1.12	0.603
	5	1.48	1.24, 1.76	<0.001	0.90	0.76, 1.07	0.226
Housing quality	Scale (0–7)	1.03	0.98, 1.08	0.242	0.96	0.92, 1.00	0.079
Level of education	Less than elementary school	Ref			Ref		
	Elementary school	0.91	0.79, 1.05	0.212	0.93	0.82, 1.06	0.285
	Middle school	1.07	0.92, 1.25	0.391	0.88	0.76, 1.02	0.087
	High school	1.23	0.99, 1.52	0.057	0.75	0.60, 0.93	0.008
	Vocational college/university	1.21	0.92, 1.58	0.181	0.74	0.54, 1.01	0.058
Smoking status	Nonsmoker	Ref			Ref		
	Current smoker	0.96	0.83, 1.10	0.524	1.24	1.08, 1.42	0.002
Alcohol intake	None	Ref			Ref		
	Once per month	0.84	0.68, 1.05	0.119	0.95	0.78, 1.17	0.648
	More than once per month	0.95	0.84, 1.09	0.469	1.19	1.05, 1.35	0.007

Table 6 shows fixed‐effects associations between province‐level variables and each outcome (Models 4–6). GRP per capita was significantly associated with lower odds of reported depression symptoms (OR: 0.86, 95% CI: 0.81, 0.92, *p* < 0.001) and IADL impairment (OR: 0.94, 95% CI: 0.87, 1.00, *p* = 0.049), and higher odds of overweight (OR: 1.01, 95% CI: 1.00, 1.02, *p*= 0.039), but not other outcomes (Model 4). Doctor density was associated with lower odds of depression symptoms (OR: 0.54, 95% CI: 0.40, 0.75, *p* = 0.002) (Model 5). After mutual adjustment, only GRP per capita was found to have a significant association with the health outcomes: there was a borderline significant association between GRP per capita and lower odds of depression symptoms, and a significant association between GRP per capita and lower odds of IADL impairment (Model 6).

**Table 6:** Summary of analyses of between‐province variance and area level effects for depression, functional disability, instrumental activities of daily living (IADL) impairment, overweight, and lung function impairment outcomes

Model	Measures of variation and clustering	Depression symptoms (CES‐D‐10)	Limitation in physical functioning	IADL impairment	Overweight	Lung function impairment
Empty model, full sample (Model 1)	Sample size	12,670	15,255	15,255	16,377	10,469
	Area‐level variance	0.22	0.12	0.09	0.24	0.13
	Intraclass‐correlation coefficient (ICC)	0.065	0.036	0.027	0.068	0.039
	Median odds ratio (MOR)	1.58	1.39	1.33	1.59	1.41
Empty model, analytic sample (Model 2)	Sample size	8204	8874	8874	8874	6692
	Area‐level variance	0.20	0.08	0.09	0.22	0.14
	proportional change in variance (PCV) (%)	0.058	0.023	Ref	Ref	Ref
	ICC	1.53	1.30	0.026	0.064	0.040
	MOR	Ref	Ref	1.33	1.57	1.42
Model with individual‐level variables, analytic sample (Model 3)	Sample size	8204	8874	8874	8874	6692
	Area‐level variance	0.11	0.07	0.09	0.11	0.12
	ICC (rescaled)	0.032	0.021	0.025	0.032	0.036
	MOR	1.36	1.28	1.32	1.38	1.39
	PCV (%)	46.5	9.4	3.3	50.0	10.8
Model 3 + GRP per capita, analytic sample (Model 4)	Sample size	8204	8874	8874	8874	6692
	Gross regional product (GRP) per capita OR (95% confidence interval [CI]), *p*	0.86 (0.81, 0.92), *p* < 0.001	0.97 (0.91, 1.03), *p* = 0.258	0.94 (0.87, 1.00), *p* = 0.049	1.01 (1.00, 1.02), *p* = 0.039	0.98 (0.91, 1.07), *p* = 0.674
	Area‐level variance	0.06	0.06	0.06	0.11	0.12
	ICC (rescaled)	0.017	0.019	0.019	0.029	0.034
	MOR	1.25	1.27	1.27	1.35	1.39
	PCV (%)	72.3	17.1	28.2	55.4	14.1
Model 3 + doctor density, analytic sample (Model 5)	Sample size	8204	8874	8874	8874	6692
	Doctor density OR (95% CI), *p*	0.54 (0.40, 0.75), *p* = 0.002	0.98 (0.76, 01.27), *p* = 0.908	0.95 (0.70, 1.28), *p* = 0.716	1.28 (0.94, 1.75), *p* = 0.119	0.84 (0.61, 1.15), *p* = 0.279
	Area‐level variance	0.06	0.07	0.08	0.11	0.11
	ICC (rescaled)	0.019	0.020	0.025	0.032	0.032
	MOR	1.27	1.28	1.32	1.37	1.37
	PCV (%)	67.9	9.64	5.5	50.84	20.4
Model 3 + GRP per capita and doctor density, analytic sample (Model 6)	Sample size	8204	8874	8874	8874	6692
	GRP per capita OR (95% CI), *p*	0.99 (0.98, 0.99), *p* = 0.052	0.99 (0.98, 1.00), *p* = 0.114	0.99 (0.98, 1.00), *p* = 0.014	1.01 (1.00, 1.02), *p* = 0.181	1.00 (0.99, 1.02), *p* = 0.617
	Doctor density OR (95% CI), *p*	0.77 (0.49, 1.27), *p* = 0.263	1.25 (0.85, 1.83), *p* = 0.263	1.40 (0.92, 2.16), *p* = 0.115	1.01 (0.62, 1.60), *p* = 0.996	0.76 (0.46, 1.24), *p* = 0.277
	Area‐level variance	0.06	0.06	0.06	0.10	0.11
	ICC (rescaled)	0.017	0.18	0.019	0.029	0.032
	MOR	1.26	1.26	1.27	1.35	1.37
	PCV (%)	71.7	21.5	27.4	55.4	19.27

### 3.3 Random‐effects

Random‐effects area‐level variance parameter estimates for each health outcome are shown in Table 6. Variance parameter estimates were similar between Models 1 and 2 for all outcomes; this implies that estimates of province effects were unaffected by loss of respondents with missing covariate observations from the analytic sample.

**Figure 1:**
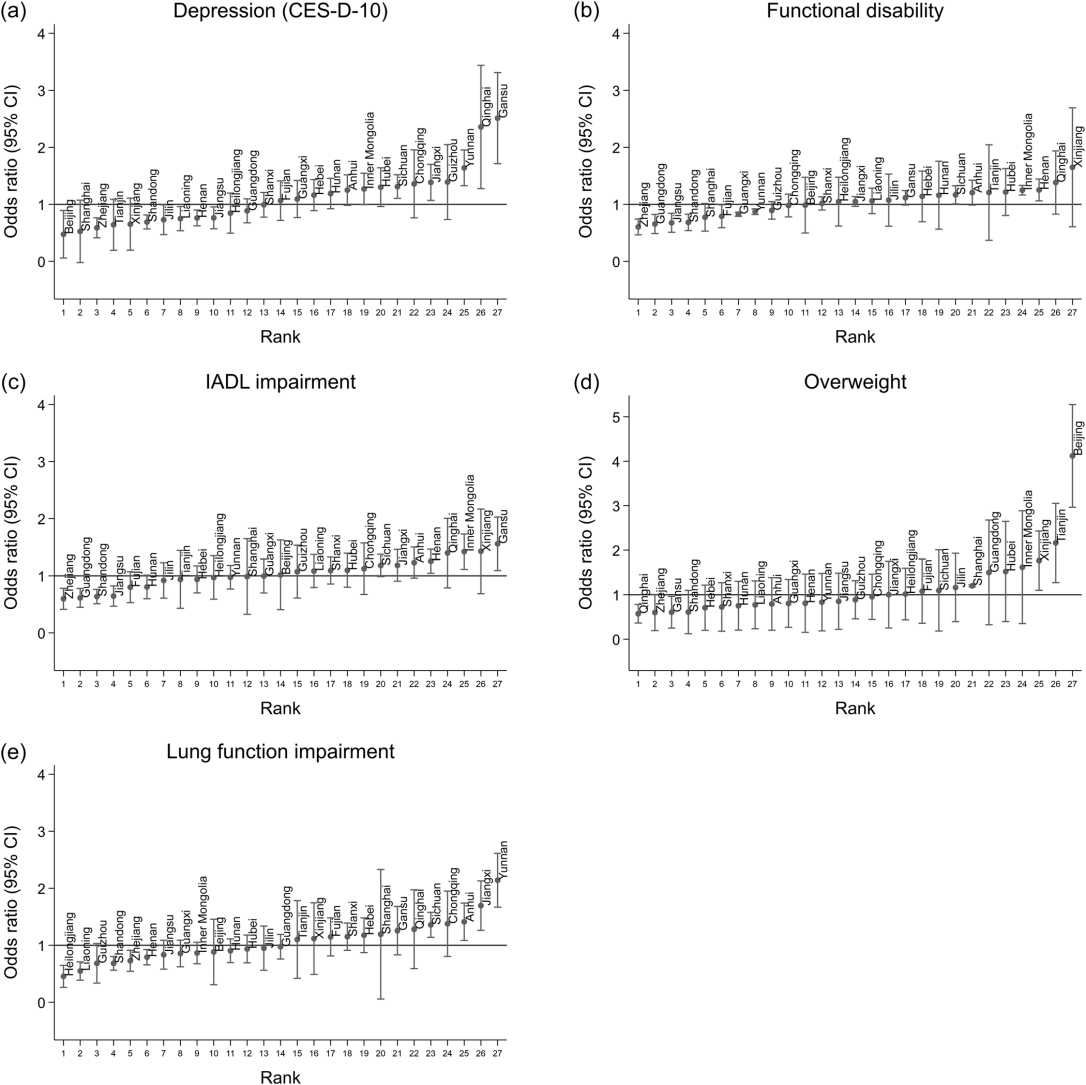
Random‐intercepts residual plots of province‐level effects for depression symptoms (CES‐D‐10), (a) limitation in physical functioning, (b) instrumental activities of daily living (IADL) impairment, (c) overweight (d) and lung function impairment (E) derived from unconditional models (Model 2) expressed as odds ratios with 95% confidence intervals

ICC estimates for Model 2 showed that province effects accounted for 5.8% of variance in depression symptoms, 6.4% in overweight, and 4.0% in lung function impairment, but only 2.3% and 2.6% of variance in limitation in physical functioning and IADL impairment in the analytic sample (Model 2). Figure 1(a–e) shows random‐effects residuals for each health outcome by province transformed into ORs with 95% CIs relative to the grand mean for the 27 province‐level units (Model 2). MORs ranged from 1.53 for depression symptoms and 1.57 for overweight to 1.30 and 1.33 for limitation in physical functioning and IADL impairment.

The results of Model 3 show random‐effects statistics after adjustment for individual‐level variables (compositional effects). PCV estimates show that these accounted for 46.5% and 50.% of between‐province variance in depression symptoms and overweight but only 9.4% and 3.3% for limitation in physical functioning and IADL impairment.

After adjustment for GRP per capita, Model 4 explained the largest proportions of between‐province variance in depression symptoms, IADL impairment, and overweight (72.3%, 28.2% and 55.4%). After adjustment for doctor density, meanwhile, Model 5 explained 67.9% and 50.8% of between‐province variance in depression symptoms and overweight, but only 9.6% and 5.5% of variance for limitation in physical functioning and IADL impairment. When models mutually adjusted for GRP per capita and doctor density (Model 6), PCV estimates for depression symptoms, limitation in physical functioning and IADL impairment were 71.7%, 21.5%, and 27.4%.

## 4 DISCUSSION

After mutual adjustment for GRP per capita and doctor density, only GRP per capita both independently predicted depression symptom outcomes and limitation in physical functioning. Within‐province differences (Model 2) still explained the majority of variance across all outcomes (93.6%–97.4%). The scale of province‐level differences, as shown by MORs, was still large across all outcomes. The impact of residual between‐province residual heterogeneity on depression symptom outcomes (MOR: 1.36) is comparable to that of being divorced, separated, or widowed (OR: 1.33, 95% CI: 1.15, 1.53) (Model 3). Although 46.5% and 50.0% of between‐province variance in depression symptoms and overweight outcomes were explained by compositional effects (Model 3), PCVs were ≤10.6% for all other outcomes. GRP per capita was important in explaining between‐province variation in depression symptom outcomes (Model 4, PCV: 72.3%).

To my knowledge, this study is the first to quantify the relevance of between‐province heterogeneity in health outcomes in China, and to estimate the degree to which these are explained by province‐level variables. Its methods present a new approach to investigating health inequalities at the national and subnational levels. The results highlight the wide inequalities among people aged ≥50 years in multiple health outcomes between Chinese provinces as found in previous studies [10, 13, 14, 15]. As Zhou et al. [6] conclude: “China in epidemiological terms is not one nation, but five: rapid transitions are occurring in all of them, but the most important health problems and the challenges imposed on the health system by demographic and epidemiological change are different.”

The results underscore that there is relatively limited potential for reduction in between‐province health inequalities through ensuring more equal distribution of healthcare personnel, with GRP per capita being more important in explaining between‐province differences in health outcomes (particularly for depression and IADL impairment outcomes). However, availability and distribution of qualified healthcare personnel can be improved through expansion of training, standardization of credentials and specific initiatives such as exchange programmes to increase equity between provinces [36, 37].

### 4.1 Strengths and limitations

Strengths of the study include its large sample size and CHARLS’ representativeness of the Chinese population. The available data provide near‐complete coverage of Chinese provinces, in addition to a wide range of health outcomes encompassing psychosocial and physical functioning, and objective measures of BMI and lung function.

CHARLS only samples official residents with valid Hukou status and excludes individuals residing in institutions such as nursing homes, (the latter represent only a small proportion of older people in China) [18]. Non‐Hukou residents are more likely to reside in provinces with higher GRP per capita, and may experience disparities in health and access to healthcare compared with permanent legal (Hukou‐holding) residents [3]. This may have undermined representativeness of province‐level samples, and inflated GRP per capita estimates for wealthier provinces [38]. Doctor density does not account for distribution of personnel across primary and secondary care facilities or numbers of specialists.

The results of this study do not imply causation as to the province‐level determinants of the health outcomes analyzed but provides a description of the degree to which they are explained by the former. If causal associations exist, these may be mediated by other factors; for example, the association between province‐level GRP and overweight may be mediated by factors such as nutrition and sedentary lifestyle.

## 5 CONCLUSION

Ongoing reforms are targeted at equalizing investment and public services across provinces [7], in addition to achieving universal and equitable coverage of basic healthcare for all Chinese citizens [35]. As all provinces of China complete their epidemiological transition, tackling the noncommunicable disease burden will be key to reducing both social and health inequalities [4]. Success in reducing between‐province health inequalities requires a coordinated health policy approach across national and province‐level governments. Localized approaches to healthcare delivery are needed to address different provinces’ diverse challenges, however [6].

## AUTHOR CONTRIBUTIONS

**Sol Richardson**: Conceptualization (lead); formal analysis (lead); investigation (lead); methodology (lead); writing – original draft (lead); writing – review and editing (lead). **Zhihui Li**: Writing – review and editing (supporting).

## CONFLICT OF INTEREST

The authors declare no conflict of interest.

## ETHICS STATEMENT

Ethical approval for all the CHARLS waves was granted from the Institutional Review Board at Peking University. The IRB approval number for the main household survey, including anthropometrics, is IRB00001052‐11015; the IRB approval number for biomarker collection was IRB00001052‐11014.

## INFORMED CONSENT

All CHARLS participants who provided data for this study provided written informed consent at the time of participation.

## Data Availability

The CHARLS data employed in this study are available free of charge to registered users through the CHARLS website at http://charls.pku.edu.cn/en.

## Footnotes

1 This this age cutoff was selected as it corresponds to the minumim age for inclusion in comparable panel studies (such as the Health and Retirement Study (HRS) and the English Longitudinal Study of Ageing (ELSA), is consistent with typical definitions of an “older person” employed in previous studies, and, in China, is the minimum statutory retirement age (for women).

2 Depression symptom outcomes were measured using the 10‐item Center for Epidemiological Studies Depression Scale (CES‐D‐10). This instrument was based on a 10‐item scale comprising symptoms includeing feeling “bothered,” “trouble concentrating,” “depressed,” “effort,” “fearful,” “restless sleep,” “hard to get going,” “hopeful,” and “happy” within the last week. Each item was scored on a four‐point scale ranging from 0 (“rarely or none of the time”) to 3 (“most or all of the time”), and the former two positively worded items were reverse‐coded before calculating summary scores. Summary scores ranged from 0 to 30 and cutoff score of 10 was used to define probable depressive cases.

3 Respondents were categorized as having a limitation in physical functioning if they self‐reported difficulties in performing any of the following tasks: sitting, climbing stairs, kneeling, reaching or extending, lifting objects, picking up objects. This variable was not based on a validated scale but has been employed in previous studies on functional abilities of older people. While some studies^25^ refer to these as “limitation in physical functioning,” other studies have also used the term “limitation in mobility” or “limitation in mobility activities”.^26^, ^27^

4 Respondents were defined as having an IADL impairment if they reported problems carrying out any of the following: managing finances, managing transportation, shopping, (hot) meal preparation, household chores, communication, and managing medications.

5 Respondents' peak expiratory flow (PEF), defined using the highest of three measures taken with a peak flow meter during the nurse visit, was compared with their expected value based on their gender, age, and height calculated using the equations below (see Nunn and Gregg [22]):
$\mathrm{PEF}(\mathrm{male})=e^{\left((0.544*\ln(\mathrm{Age}))-(0.0151*\mathrm{Age})-(74.7/\mathrm{Height})+5.48\right)}$

$\mathrm{PEF}(\mathrm{female})=e^{\left((0.376*\ln(\mathrm{Age}))-(0.012*\mathrm{Age})-(58.8/\mathrm{Height})+5.63\right)}$
Respondents whose actual PEF was <70% of their predicted value were categorized as having lung function impairment. This cutoff represents a highly sensitive measure of severe COPD.

6 CHARLS Wave 2 respondents were invited to self‐report their type of health insurance coverage from a list of 10 options. In this study, respondents were categorized by their type of health insurance coverage using the four categories below. Categorization was hierarchical, and respondents who reported having more than one type of health insurance coverage were assigned to the highest (lowest‐numbered) category: 1. Government scheme, enhanced coverage: Government employee medical insurance (公费医疗), urban employee medical insurance (职工医保) 2. Government scheme, basic coverage: New cooperative medical insurance (新型农村合作医疗), rural and urban resident medical insurance (城乡居民基本医疗保险) 3. Private or other: Any other insurance scheme not included in the questionnaire 4. Uninsured: No membership of any health insurance scheme reported.

## References

[1] Lin JY . China's growth miracle in the context of Asian transformation. Helsinki: World Institute for Development Economics Research (UNU‐WIDER); 2018. 10.35188/UNU-WIDER/2018/534-3

[2] Li S , Xu Z . The trend of regional income disparity in the People's Republic of China. Tokyo: Asian Development Bank Institute; 2008.

[3] Jain‐Chandra S , Khor H , Mano R , Schauer J , Wingender W , Zhuang J . Inequality in China‐trends, drivers and policy remedies. Washington, DC: International Monetary Fund; 2018.

[4] Yang G , Kong L , Zhao W , Wan X , Zhai Y , Chen LC , et al. Emergence of chronic non‐communicable diseases in China. Lancet. 2008;372(9650):1697–705. 10.1016/S0140-6736(08)61366-5 18930526

[5] Campbell TC , Junshi C , Brun T , Parpia B , Yinsheng Q , Chumming C , et al. China: from diseases of poverty to diseases of affluence. policy implications of the epidemiological transition. Ecol Food Nutr. 1992;27(2):133–44. 10.1080/03670244.1992.9991235

[6] Zhou M , Wang H , Zhu J , Chen W , Wang L , Liu S , et al. Cause‐specific mortality for 240 causes in China during 1990–2013: a systematic subnational analysis for the global burden of disease study 2013. Lancet. 2016;387(10015):251–72. 10.1016/S0140-6736(15)00551-6 26510778

[7] Fan S , Kanbur R , Zhang X . China's regional disparities: experience and policy. Rev Dev Finance. 2011;1(1):47–56. 10.1016/j.rdf.2010.10.001

[8] Chou WL , Wang Z . Regional inequality in China's health care expenditures. Health Econ. 2009;18(suppl 2):S137–46. 10.1002/hec.1511 19548323

[9] Zhou K , Zhang X , Ding Y , Wang D , Lu Z , Yu M . Inequality trends of health workforce in different stages of medical system reform (1985–2011) in China. Hum Resour Health. 2015;13:94. 10.1186/s12960-015-0089-0 26645960 PMC4673776

[10] Qin X , Wang S , Hsieh CR . The prevalence of depression and depressive symptoms among adults in China: estimation based on a national household survey. China Econ Rev. 2018;51:271–82. 10.1016/j.chieco.2016.04.001

[11] Easterlin RA , Morgan R , Switek M , Wang F . China's life satisfaction, 1990‐2010. Proc Natl Acad Sci. 2012;109(25):9775–80. 10.1073/pnas.1205672109 22586096 PMC3382525

[12] Liang Y , Welmer A‐K , Möller J , Qiu C . Trends in disability of instrumental activities of daily living among older Chinese adults, 1997‐2006: population based study. BMJ Open. 2017;7(8):e016996. 10.1136/bmjopen-2017-016996 PMC5724119 28851795

[13] He Y , Pan A , Wang Y , Yang Y , Xu J , Zhang Y , et al. Prevalence of overweight and obesity in 15.8 million men aged 15–49 years in rural China from 2010 to 2014. Sci Rep. 2017;7(1):5012. 10.1038/s41598-017-04135-4 28694524 PMC5504069

[14] Fang L , Gao P , Bao H , Tang X , Wang B , Feng Y , et al. Chronic obstructive pulmonary disease in China: a nationwide prevalence study. Lancet Res Med. 2018;6(6):421–30. 10.1016/S2213-2600(18)30103-6 PMC7185405 29650407

[15] Evandrou M , Falkingham J , Feng Z , Vlachantoni A . Individual and province inequalities in health among older peoplein China: evidence and policy implications. Health Place. 2014;30:134–44. 10.1016/j.healthplace.2014.08.009 25262491

[16] Feng Z , Wang WW , Jones K . A multilevel analysis of the role of the family and the state in self‐rated health of elderly Chinese. Health Place. 2013;30:148–56. 10.1016/j.healthplace.2013.07.001 23906587

[17] Richardson S , Carr E , Netuveli G , Sacker A . Country‐level welfare‐state measures and change in wellbeing following work exit in early old age: evidence from 16 European countries. Int J Epidemiol. 48(2):389–401. 10.1093/ije/dyy205 PMC6469302 30277529

[18] Zhao Y , Hu Y , Smith JP , Strauss J , Yang G . Cohort profile: the China Health and Retirement Longitudinal Study (CHARLS). Int J Epidemiol. 2014;43(1):61–8. 10.1093/ije/dys203 23243115 PMC3937970

[19] Chen H , Mui AC . Factorial validity of the Center for Epidemiologic Studies Depression Scale short form in older population in China. Int Psychogeriatr. 2014;26(1):49–57. 10.1017/S1041610213001701 24125553

[20] Andresen EM , Malmgren JA , Carter WB , Patrick DL . Screening for depression in well older adults: evaluation of a short form of the CES‐D. Am J Prev Med. 1994;10(2):77–84. 10.1016/S0749-3797(18)30622-6 8037935

[21] Zhang W , O'Brien N , Forrest JI , Salters KA , Patterson TL , Montaner JSG , et al. Validating a shortened depression scale (10 item CES‐D) among HIV‐positive people in British Columbia, Canada. PLoS One. 2012;7(7):e40793. 10.1371/journal.pone.0040793 22829885 PMC3400644

[22] Tobias DK , Hu FB . Commentary: obesity and mortality in China: the shape of things to come. Int J Epidemiol. 2012;41(2):481–3. 10.1093/ije/dys031 22407861

[23] Nunn AJ , Gregg I . New regression equations for predicting peak expiratory flow in adults. BMJ. 1989;298(6680):1068–70. 10.1136/bmj.298.6680.1068 2497892 PMC1836460

[24] Perez‐Padilla R , Vollmer WM , Vázquez‐García JC , et al. Can a normal peak expiratory flow exclude severe chronic obstructive pulmonary disease? Int J Tuberc Lung Dis. 2009;13(3):387393. PMC3334276 19275802

[25] Seidel D , Brayne C , Jagger C . Limitations in physical functioning among older people as a predictor of subsequent disability in instrumental activities of daily living. Age Ageing. 2011;40(4):463–9. 10.1093/ageing/afr054 21609999 PMC3114622

[26] Gale CR , Cooper C , Sayer AA . Prevalence of frailty and disability: findings from the English Longitudinal Study of Ageing. Age Ageing. 2015;44(1):162–5. 10.1093/ageing/afu148 25313241 PMC4311180

[27] Bloomberg M , Dugravot A , Landré B , et al. Sex differences in functional limitations and the role of socioeconomic factors: a multi‐cohort analysis. Lancet Healthy Longev. 2021;2(12):E780–90. 10.1016/S2666-7568(21)00249-X 34901907 PMC8636280

[28] National Bureau of Statistics . China 2014 Statistical Yearbook. Beijing: NBS; 2014.

[29] OECD . PPPs and exchange rates. OECD National Accounts Statistics. Paris: Organisation for Economic Co‐operation and Development; 2015.

[30] StataCorp . Stata Statistical Software: Release 14. College Station, TX: StataCorp LP; 2015.

[31] Goldstein H , Browne W , Rasbash J . Partitioning variation in multilevel models. Understand Statist. 2002;1:223–31. 10.1207/S15328031US0104_02

[32] Hox JJ . Multilevel analysis: techniques and applications. Mawah, NJ: Earlbaum; 2002.

[33] Merlo J , Chaix B , Ohlsson H , et al. A brief conceptual tutorial of multilevel analysis in social epidemiology: using measures of clustering in multilevel logistic regression to investigate contextual phenomena. J Epidemiol Commun Health. 2006;60(4):290–7. 10.1136/jech.2004.029454 PMC2566165 16537344

[34] Merlo J , Chaix B , Yang M , Lynch J , Rastam L . A brief conceptual tutorial on multilevel analysis in social epidemiology: investigating contextual phenomena in different groups of people. J Epidemiol Commun Health. 2005;59(9):729–36. 10.1136/jech.2004.023473 PMC1733145 16100308

[35] Enzmann D , Kohler U . MERESC: Stata module to rescale the results of mixed nonlinear probability models. Statistical Software Components, Boston College Department of Economics; 2012.

[36] Wang T , Zeng R . Addressing inequalities in China's health service. Lancet. 2015;386(10002):1441. 10.1016/S0140-6736(15)00402-X 26466037

[37] Anand S , Fan VY , Zhang J , Zhang L , Ke Y , Dong Z , et al. China's human resources for health: quantity, quality, and distribution. Lancet. 2008;372(9651):1774–81. 10.1016/S0140-6736(08)61363-X 18930528

[38] Chan KW , Wang M . Remapping China's regional inequalities, 1990‐2006: a new assessment ofde factoandde JurePopulation data. Eurasian Geogr Econ. 2008;49(1):21–55. 10.2747/1539-7216.49.1.21

